# 

**Published:** 1892-09-03

**Authors:** 


CONTENTS.

| | PAGE |
|---|---|
| **First Lines of Defence** | 369 |
| **Passing Topics.**—Hospitals Closed for Repairs | 370 |
| **The Doctor's Armchair.**—Facts about Cholera | 371 |
| **The History and Capabilities of Herbal Simples.** By W. T. Fernie, M.D.—Garlic and the Onion | 372 |
| **Disease the Monitor** | 373 |
| **Everybody's Page** | 374 |
| **Annotations.**—Jewish and Gentile Children—The Harmless, necessary Tart—An Imperial Medical "Home and Club" | 375 |
| **Notes and News** | 376 |
| **Scraps and Gleanings** | 377 |
| **Edtior's Letter-Box.**—The Cost of Out-Patients at Special Hospitals | 380 |
| **Quackery and Christianity** | 380 |
| **The Practitioner's Mirror and Retrospect**—Medicine.—On Some of the Relations Between Chorea and Rheumatism—The Treatment of Lupus Vulgaris | 378 |
| Surgery.—Tumours of the Bladder: Their Pathology and Diagnosis | 379 |
| **The Institutional Workshop**—Hospital Administration II.—Special Hospitals | 381 |
| Construction and Management.—The Military (Alexander) Hospital. Warsaw | 382 |
| **The Cure of Poverty.**—An Account of the North Kensington Friendly Workers among the Poor. By Mrs. C. Furley-Smith (*continued*) | 383 |
| **The Student of Medicine.**—Appointments—Vaccancies—Pass Lists. | |

EXTRA SUPPLEMENT.—THE NURSING MIRROR.

| | |
|---|---|
| **En Passant.**—Practical Points—The Fever Hospitals—Fever Nurses—A New Home for Invalids at Cannes—The Prospects of Southampton Nurses—The Kitchen at Plaistow, E. | clxi |
| **Lectures for Asylum Attendants.** By William Harding, M.B. I.—Introductory (*continued*) | clxii |
| **How to Procure Nurses for India Locally** | clxiii |
| **Appointments** | clxiii |
| **For Reading to the Sick.**—"Loss or Gain?" | clxiii |
| **Nursing Homes.**—The Hampshire Nurses' Institute | clxiv |
| **Cholera.** From the Nurse's Point of View | clxiv |
| **Everybody's Opinion.**—Male Nurses—Cambridge Home and Training School for Nurses—Private Nurses | clxv |
| **Visitors in General Hospital Wards** | clxvi |
| **Some Australian Experiences** | clxvii |
| **Presentation—Notes and Queries** | clxvii |
| **"The Flowers of Sleep"** | clxviii |

